# Retraction notice to “Selective mapping of Brain COX-1 with [11 C]PS13: Pharmacokinetic evidence from human PET imaging” [IBRO Neurosci. Rep. 18 (2025) 657–662]

**DOI:** 10.1016/j.ibneur.2026.08.004

**Published:** 2026-08-11

**Authors:** Kiana Orangi, Kimiya Batebi, Farnoosh Vosough, Mahdiyeh Nozad Varjovi, Fatemeh Salehian, Sahar Mesbah, Mehrnaz Salahi, Sajjad Hajihosseini, Mohammad Yousef Fazel, Saman Zaman, Reza Hossein Zadeh, Alaleh Alizadeh, Mahsa Asadi Anar, Niloofar Deravi

**Affiliations:** a Faculty of Medicine, Iran University of Medical Sciences, Tehran, Iran; b Doctor of Pharmacy (Pharm.D.), School of Pharmacy, Islamic Azad University of Medical Sciences, Tehran, Iran; c Student Research Committee, School of Medicine, Iran University of Medical Sciences, Tehran, Iran; d Student Research Committee, School of Pharmacy, Tabriz University of Medical Sciences, Tabriz, Iran; e Student Research Committee, School of Medicine, Shahid Beheshti University of Medical Sciences, Tehran, Iran; f Student Research Committee, Faculty of Pharmacy, Shahid Sadoughi University of Medical Sciences, Yazd, Iran; g Novel Drug Delivery Systems Research Centre, Department of Pharmaceutics, School of Pharmacy and Pharmaceutical Sciences, Isfahan University of Medical Sciences, Isfahan, Iran; h Student Research Committee, Faculty of Pharmacy, Tehran University of Medical Sciences, Tehran, Iran; i School of Pharmacy, Shahid Beheshti University of Medical Sciences, Tehran, Iran; j Ahvaz Jondishapur University of Medical Sciences, Ahvaz, Iran; k Student Research Committee, Faculty of Medicine, Mashhad University of Medical Sciences, Tehran, Iran; l Students Research Committee, Faculty of Medicine, Mashhad Branch, Islamic Azad University, Mashhad, Iran; m College of Medicine, University of Arizona, Tucson, AZ, USA

This article has been retracted: please see Elsevier Policy on Article Withdrawal (https://www.elsevier.com/about/policies/article-withdrawal).

This article has been retracted at the request of the Editors.

Following concerns raised regarding the published article, the Editorial Board conducted an initial assessment and sought the advice of an independent external expert. Based on this review, the Editors identified concerns relating to data provenance, copyright, data ownership, and publication ethics.

Specifically, the Editorial Board found that:

(i) The provenance of the datasets, parameters, and results presented in the article could not be established with sufficient clarity. In particular, the OpenNeuro dataset cited by the authors did not contain the celecoxib or aspirin data on which key analyses and conclusions in the article were based.
(ii) The authors' description of the nature and scope of the work evolved across successive clarification letters, i.e., from an original contribution based on PET imaging reanalysis, to a “secondary pharmacokinetic reanalysis,” and subsequently to a “meta-modelling perspective” and “cross-study synthesis.” Consequently, the Editorial Board was unable to determine with confidence which aspects of the work represented original analyses conducted by Orangi et al. and which were derived from previously published materials.
(iii) Statements in the article regarding ethical approval, participant recruitment, informed consent, and study conduct were not consistent with the information subsequently provided by the authors regarding their role in the work.

In light of these concerns, the Editorial Board concluded that the reliability of the findings and conclusions presented in the article could not be confirmed. The authors were invited to respond to these concerns but did not provide sufficient evidence to resolve them. Accordingly, the Editors have lost confidence in the integrity of the article and are retracting it.

